# Correlation between nasal resistance and different acoustic rhinometry parameters in children and adolescents with and without allergic rhinitis

**DOI:** 10.5935/1808-8694.20120038

**Published:** 2015-10-20

**Authors:** Gustavo Falbo Wandalsen, Aline Inês Mendes, Dirceu Solé

**Affiliations:** a PhD in Sciences, UNIFESP-EPM (Adjunct Professor in the Allergy, Immunology, and Rheumatology Course at UNIFESP-EPM); b MSc in Sciences, UNIFESP-EPM (Associate Researcher in the Allergy, Immunology, and Rheumatology Course at UNIFESP-EPM); c Associate Professor, UNIFESP-EPM (Professor in the Allergy, Immunology, and Rheumatology Course at UNIFESP-EPM). Allergy, Clinical Immunology and Rheumatology Program - Department of Pediatrics - Federal University of São Paulo (UNIFESP-EPM)

**Keywords:** nasal obstruction, rhinitis, rhinomanometry, rhinometry, acoustic

## Abstract

Acoustic rhinometry and rhinomanometry are important tests used to assess nasal function. The degree to which the parameters of these tests are correlated is yet to be established.

**Objective:**

This paper aimed to study the correlations between nasal resistance (NR) and acoustic rhinometry parameters in children and adolescents with allergic rhinitis and controls.

**Method:**

Twenty patients with allergic rhinitis and 20 controls were enrolled. NR, volumes (V4, V5, V2-5), and minimal cross-sectional areas (MC1, MC2) were measured in three moments: baseline, after induction of nasal obstruction and after topical decongestant administration.

**Results:**

Patients with allergic rhinitis had significant correlation between NR and all volumes (V5: r = -0.60) and with MC2. Among controls, MC1 was the parameter with the strongest correlation with NR at baseline (r = -0.53) and after decongestant administration. In the combined analysis, V5 had the highest correlation coefficients at baseline (r = -0.53), after obstruction (r = -0.58) and after decongestant (r = -0.46).

**Conclusions:**

Our data showed that NR and acoustic rhinometry parameters have negative and significant correlations. Nasal volumes are, in general, better correlated than minimal cross-sectional areas. V5 was the parameter with the highest correlation in the rhinitis group and in the combined analysis.

## INTRODUCTION

Rhinomanometry is one of the most widely studied, employed, and standardized tests used to assess nasal function. Dynamic measurements of the ratios between airflow and pressure levels in the nasal cavity can be obtained from rhinomanometry to compute nasal resistance^1^. It has been shown that nasal resistance (NR) is a reliable parameter to monitor the effect of drug therapy and follow nasal provocation tests^2^.

Among the other available tests, acoustic rhinometry (AR) appears to hold significant promise. This is a relatively new test which allows one to assess nasal geometry using a device - the acoustic rhinometer - that emits and captures sound waves on the entrance of the nasal cavity to map its anatomy, measure volumes, and cross sectional areas at various points^3^. Studies have shown that AR is a reliable and reproducible method when it comes to measuring the nasal volumes of children and adults^4^. Due to its properties, AR has been used to study the definitions concerning surgery indications of patients with upper airway anatomic disturbances^5^, to assess the effect of drugs used to treat allergic rhinitis^6^ and to enhance the understanding of nasal physiology^7^.

These two tests, however, measure variables of different natures. While rhinomanometry dynamically calculates a physiological variable (NR) connected to nasal breathing, AR statistically measures nasal cavity volumes and cross sectional areas. Both tests have been independently validated, but information available on the correlations between them is scarce. The extent to which one test can be correlated to the other, the AR parameters that can be compared against NR, and the possible variations between adults and children or even between patients with varying degrees of nasal obstruction are yet to be defined.

This study aimed to assess the correlations between NR and various parameters related to volumes and cross sectional areas measured using AR in children and adolescents with persistent allergic rhinitis and healthy controls.

## METHOD

### The patients

The allergic rhinitis group was made up of children and teens aged between six and 18 years followed up regularly at a specialized clinic. All subjects had been diagnosed with persistent allergic rhinitis for at least a year according to the ARIA^8^ initiative precepts and positive skin allergy tests (mean papule diameter greater than 3 mm)^9^ for at least one inhaled allergen (*D.pteronyssinus, D.farinae, Blomia tropicalis*, dog epithelium, cat epithelium, *Periplaneta americana, Blatella germanica*, mix of fungi, pollen mix [IPI-ASAC, Brazil]). Subjects with significant upper airway anatomic defects (deviated septum [anterior rhinoscopy] and enlarged adenoids [cavum x-ray]), individuals on systemic or nasal steroids for the past 30 days, and patients with history of upper airway infection within the last 30 days were excluded.

Children and adolescents within the same age range made up the control group. The subjects in this group had no history of rhinitis and other atopic diseases, did not present significant alteration in the nasal fossae on anterior rhinoscopy, and were negative for allergy for the same set of inhaled allergens used to test case group individuals.

### Nasal function assessment

Two consecutive tests were used to evaluate nasal function. All tests were carried out with the subjects seated with their heads on a neutral position and after they had waited for 20 minutes to get used to the controlled room temperature (20°C to 25°C) and humidity (50%) conditions. AR was performed with an SRE 2000 acoustic rhinometer (Rhinometrics, Denmark) in accordance with published recommendations^1^. The following parameters were assessed: volume of the proximal portion of the nasal cavity from 0 to 4.0 cm (V4), between 0 and 5.0 cm (V5), of the segment between 2.0 and 5.0 cm (V2-5), and the smaller cross sectional area in the segments between 0 and 2.2 cm (MC1) and 2.2 and 5.4 cm (MC2). The size of each nostril was assessed and analyzed separately.

NR was measured through active anterior rhinomanometry (AAR) with the same device used to perform AR. NR (inhalation) was measured at 75 Pa by the same examiner three times. Only measurements with variation under 10% were accepted.

The parameters were captured at three different stages:

1. Baseline: after acclimation to the test room and before subjects were given medication.
2. Obstruction: after the completion of the nasal provocation testing, after an increase of at least 100% on baseline nasal resistance, after delivery of solutions with different levels of histamines (0.12; 0.25; 0.5; 1.0; 2.0; 4.0 & 8.0 mg/ml, IPI-ASAC Brazil).
3. Unobstructed: 10 minutes after giving topical decongestants to subjects (three drops of oxymetazoline [0.5mg/ml] on each nostril).

Assessments were carried out sequentially on the same day. The first was the baseline test, followed by the tests done after nasal obstruction had been induced, and lastly by the tests when nasal obstruction was no longer present (after delivery of decongestants).

The correlations between the variables were analyzed through Spearman's rank correlation ratio using software package SPSS 14.0. A significance level of 5% was defined to reject the null hypothesis.

This study was approved by the Research Ethics Committee at UNIFESP-EPM (permit n^o^ 0705/04). Informed consent was obtained from the guardians of the participants before the tests were carried out.

## RESULTS

Three of the 25 patients selected into the allergic rhinitis group were excluded due to technical difficulties while attempting to perform the tests. One failed to cooperate and another had upper airway infection at the time of the test. Similarly, four of the 24 controls were excluded, two for technical difficulties and two for having acute upper airway infection. Therefore, the groups had 20 subjects each.

Table 1 features the correlation ratios between NR values and AR parameters for patients in the allergic rhinitis group. In general terms, the correlations between NR and volume were stronger than those seen for cross sectional areas. There was no statistically significant correlation between MC1 and RN at any of the three test stages. V5, V4, and V2-5 were significantly correlated on all three stages.

**Table 1:** Spearman's correlation ratios (r) between nasal resistance (NR) and parameters obtained from acoustic rhinometry in a group of children and adolescents with allergic rhinitis assessed in the baseline stage, after induced nasal obstruction, and after decongestant administration.

RN *versus*	Baseline		Nasal Obstruction		Decongestant	
	r	*P*	r	*P*	r	*P*
V5	-0.60	< 0.001	-0.68	< 0.001	-0.49	0.001
V4	-0.49	0.001	-0.48	0.001	-0.51	< 0.001
V2-5	-0.58	< 0.001	-0.69	< 0.001	-0.54	< 0.001
MC1	-0.06	0.3	0.03	0.4	-0.08	0.3
MC2	-0.44	0.002	-0.61	< 0.001	-0.43	0.003

V5: volume from 0 to 5.0 cm into the nasal cavity; V4: volume from 0 to 4.0 cm into the nasal cavity; V2-5: nasal cavity volume in the segment between 2.0 and 5.0 cm; MC1: smaller cross sectional area between 0 and 2.2 cm; MC2: smaller cross sectional area between 2.2 and 5.4 cm.

The correlation ratios for the control group can be seen on Table 2. In this group, volumes had the poorer correlation ratios (under 0.50), with V2-5 featuring the poorest correlation with NR. Differently than what was seen in the allergic rhinitis group, MC1 was significantly correlated to NR in controls and stood out as the parameter with the strongest correlation in the baseline and unobstructed stages.

**Table 2:** Spearman's correlation ratios (r) between nasal resistance (NR) and parameters obtained from acoustic rhinometry in control subjects assessed in the baseline stage, after induced nasal obstruction, and after decongestant administration.

RN *versus*	Baseline		Nasal Obstruction		Decongestant	
	r	*p*	r	*p*	r	*p*
V5	-0.35	0.01	-0.49	0.001	-0.38	0.008
V4	-0.34	0.02	-0.38	0.007	-0.37	0.01
V2-5	-0.15	0.08	-0.32	0.02	-0.29	0.02
MC1	-0.53	< 0.001	-0.34	0.02	-0.54	< 0.001
MC2	-0.32	0.02	-0.54	< 0.001	-0.49	0.001

V5: volume from 0 to 5.0 cm into the nasal cavity; V4: volume from 0 to 4.0 cm into the nasal cavity; V2-5: nasal cavity volume in the segment between 2.0 and 5.0 cm; MC1: smaller cross sectional area between 0 and 2.2 cm; MC2: smaller cross sectional area between 2.2 and 5.4 cm.

Table 3 shows the correlation ratios between NR and AR parameters for case and control group subjects. Except for MC1, all parameters had a negative significant correlation with NR in all three stages. V5 was the parameter that best correlated with NR in the three test stages. On baseline conditions, the correlations of various volumes and NR were stronger than the ratios seen after nasal topical decongestant was offered.

**Table 3:** Spearman's correlation ratios (r) between nasal resistance (NR) and parameters obtained from acoustic rhinometry in case and control subjects assessed in the baseline stage, after induced nasal obstruction, and after decongestant administration.

RN *versus*	Baseline		Nasal Obstruction		Decongestant	
	r	*p*	r	*p*	r	*p*
V5	-0.53	< 0.001	-0.58	< 0.001	-0.46	< 0.001
V4	-0.47	< 0.001	-0.43	< 0.001	-0.45	< 0.001
V2-5	-0.49	< 0.001	-0.52	< 0.001	-0.40	< 0.001
MC1	-0.29	0.005	-0.15	0.09	-0.27	0.008
MC2	0.46	< 0.001	-0.58	< 0.001	-0.44	< 0.001

V5: volume from 0 to 5.0 cm into the nasal cavity; V4: volume from 0 to 4.0 cm into the nasal cavity; V2-5: nasal cavity volume in the segment between 2.0 and 5.0 cm; MC1: smaller cross sectional area between 0 and 2.2 cm; MC2: smaller cross sectional area between 2.2 and 5.4 cm.

Considering the data related to the three stages together, stronger correlations were seen with the various AR parameters (Table 4). In the allergic rhinitis group and in the total group V5 was again the parameter that best correlated with NR (r = -0.70 and r = -0.67, respectively), while MC2 was the parameter that correlated the best with NR in the control group (r = -0.66). The only non-significant correlation with NR was seen for parameter MC1 in the allergic rhinitis group.

**Table 4:** Spearman's correlation ratios (r) between nasal resistance (NR) and parameters obtained from acoustic rhinometry in the case and control groups and in the total group assessed in the baseline stage, after induced nasal obstruction, and after decongestant administration.

RN *versus*	Baseline		Nasal Obstruction		Decongestant	
	r	*p*	r	*p*	r	*p*
V5	-0.63	< 0.001	-0.7	< 0.001	-0.67	< 0.001
V4	-0.57	< 0.001	-0.61	< 0.001	-0.61	< 0.001
V2-5	-0.64	< 0.001	-0.57	< 0.001	-0.65	< 0.001
MC1	-0.61	0.04	-0.17	< 0.001	-0.36	< 0.001
MC2	-0.66	< 0.001	-0.6	< 0.001	-0.62	< 0.001

V5: volume from 0 to 5.0 cm into the nasal cavity; V4: volume from 0 to 4.0 cm into the nasal cavity; V2-5: nasal cavity volume in the segment between 2.0 and 5.0 cm; MC1: smaller cross sectional area between 0 and 2.2 cm; MC2: smaller cross sectional area between 2.2 and 5.4 cm.

## DISCUSSION

Nasal obstruction has been described as one of the most frequently reported symptoms by patients with rhinopathy. In patients with rhinitis, it has been associated to a series of complications such as sleep disorders, learning disorders, attention deficit, facial development disturbances, and sinusitis^10^, ^11^, ^12^, ^13^, ^14^. Various studies, however, have documented significant variability and inaccuracy as patients subjectively describe their symptoms when compared to objective testing done to the same end^15^, ^16^, ^17^, ^18^. Many explanations can be used to account for such discrepancy. Apparently, sensation of nasal obstruction is not determined solely by the size of the patent nasal cavity and may be affected, for instance, by variations in the ostiomeatal complex and a wide range of local stimuli^17^, ^19^. Additionally, patients with chronic nasal obstruction may get used to the condition and minimize its severity^16^.

Among the many objective methods developed to date to assess nasal function, rhinomanometry is the most studied and standardized test, and has been considered as the method of reference to analyze nasal function^17^, ^19^. In patients without obstructive upper airway involvement, NR indirectly reflects the degree of nasal mucosa inflam-mation^17^.

AR is a test used to assess the size of the nasal cavities. It has been validated against other anatomical assessment methods. In patients with nasal obstruction and controls, the volumes of the proximal portion of the nose (the first six centimeters) obtained by AR are similar to the values verified by CT and MRI scans^20^, ^21^, ^22^.

Up to this day, however, few studies have looked into the correlations between NR and nasal cavity measurements. Some studies resorted to nasal provocations to show that nasal obstruction induction increases nasal resistance and simultaneously reduces volume^23^, ^24^, ^25^. These studies, however, did not directly assess the correlations between these variables.

The data gathered in this study shows strong negative significant correlations between AR values and NR. In general terms, nasal volumes (V4, V5, and V2-5) were more significantly correlated to NR than smaller cross sectional areas (MC1 & MC2). This finding was consistently verified in the different stages of the study (baseline, after nasal obstruction induction, and after decongestant delivery; Table 3). Other authors have analyzed nasal function using nasal provocation tests and found that volume is a more reliable parameter than cross sectional areas^1^, ^26^. Nasal cavity volume seems to be more sensitive than smaller cross sectional areas to assess nasal conges-tion^27^ and monitor response to different dosages of nasal decongestants^28^.

The resistance of a tube is traditionally expected to be determined by its smaller diameter. Thus, the absence of a significant correlation between total nasal resistance and smaller cross sectional areas, as seen in some of the study's stages (nasal obstruction and after decongestant administration), was an unexpected finding. The relatively small number of patients enrolled in the study may have contributed to this surprise. When the same correlations were calculated for a larger sample considering all groups in the study, there was a significant improvement on the correlation ratios (Table 4). Qian et al.^27^ studied the ratios between resistance to airflow and volume and smaller cross sectional employing various nasal cavity models. By varying the volume of the model and keeping the smaller internal diameter fixed, these authors have shown that resistance is better correlated to volume than smaller cross sectional area.

An interesting observation risen from the analysis of the correlations between NR and smaller cross sectional areas was the discrepancy between findings in the case and control groups. More notably, MC1 was poorly correlated with NR in the rhinitis group and strongly correlated with NR in the control group (Tables 1 and 2). The anatomical differences found in the more proximal (MC1) and distal (MC2) portions of the nasal cavity may explain this finding. The nasal vestibule, located in the proximal portion of the nasal cavity, is covered by epithelium and has no erectile tissue, whereas the distal portion is covered by mucosa, which makes patients with allergic rhinitis more susceptible to nasal obstruction^27^.

The few studies that compared nasal resistance to AR parameters showed significant variability in their results. Differences in method, equipment, population, and variables selected for analysis may explain such variation. Scadding et al.^29^ correlated NR and cross sectional area findings in patients submitted to specific nasal provocation. In combined data analysis, the authors saw a significant inverse correlation between the variables (r = -0.60), as also found in our study for MC2 (r = -0.52). More recently, Zhang et al.^30^ published an assessment on the correlations between nasal resistance and volume from 0 to 6 centimeters in each nostril of 316 adult patients complaining of nasal obstruction. The authors found significant correlations both before (r = -0.43) and after the administration of decongestants (r = -0.37). The values found in this study were particularly close to ours.

Numminen et al.^31^ compared various objective nasal function tests in a group of 69 adult patients with acute viral infection. Three tests were carried out in different days and signifiant correlations with NR were found for both smaller cross sectional area and nasal volume (V2-5). Despite their statistical significance, all correlation ratios were under 0.40. According to the authors, this meant the correlations had low clinical significance. The same group of authors published another study in which the nasal function findings of 249 adult subjects without respiratory disease or complaints of nasal obstruction were analyzed^32^. This second study found different results when compared to the first, showing absence of significant correlations between NR and smaller cross sectional areas and nasal volume between 1.0 and 4.0 cm. Likewise, Taverner et al.^33^ failed to see significant correlations between NR baseline values and smaller cross sectional areas (2.2 to 5.5 cm) in 52 common cold patients.

Despite the differences, there seems to be some consensus on the significant independence observed between AR and NR variables. The correlation ratios found in our study (in the 0.5 range) support this idea. The differences in method and the varying nature of the variables can be additionally used to justify this finding.

There is still no consensus as to which nasal cavity volume is the most suitable in the AR examination of children. In adult patients, it is recommended that at least V5 be measured at all times and that V2-5 be added when nasal mucosa alterations are assessed^1^. Smaller volumes have been suggested for pediatric patients^27^. In this study we opted to assess the NR correlations with different volumes. On the baseline stage, no striking differences were found in the correlations between NR and the tested volumes (V5, V4, and V2-5; Table 3). V5 was the parameter that presented the strongest correlation with NR in patients with allergic rhinitis and in both groups considered together (Table 4).

## CONCLUSION

This study revealed a significant correlation between NR and different anatomic variables measured by AR in children and adolescents with allergic rhinitis and healthy controls. In most of the tests, nasal cavity volume was better correlated to NR than smaller cross sectional areas. V5 was the isolated parameter more significantly correlated to NR, regardless of the presence of nasal disease or nasal obstruction.
